# Serum Inhibin B and Cardiometabolic Profile in Adult Men: A Cross-Sectional Study

**DOI:** 10.3390/metabo16090639

**Published:** 2026-09-01

**Authors:** Ayça Tuzcu, Cem Kaba, Arzu Ateş, Kenan Yörük, Mustafa Yılmaz, Göksel Tuzcu

**Affiliations:** 1 Department of Biochemistry, Faculty of Medicine, Aydın Adnan Menderes University, 09100 Aydın, Türkiye; atuzcu@adu.edu.tr (A.T.); cemkaba98@gmail.com (C.K.); dr.arzulgen968@gmail.com (A.A.); kenan.yoruk@adu.edu.tr (K.Y.); mustafa.yilmaz@adu.edu.tr (M.Y.); 2 Department of Radiology, Faculty of Medicine, Aydın Adnan Menderes University, 09100 Aydın, Türkiye

**Keywords:** inhibin B, triglyceride–glucose index, atherogenic index of plasma, insulin resistance, body mass index, cardiometabolic risk, male reproductive hormones, coronary artery calcium score

## Abstract

**Background/Objectives**: The extent to which inhibin B, a marker of Sertoli cell function, is affected by cardiometabolic disturbances remains insufficiently understood. The aim of this study was to evaluate the relationships between serum inhibin B levels and the atherogenic index of plasma (AIP), triglyceride–glucose (TyG) index, HOMA-IR, METS-IR, and C-reactive protein (CRP) in adult men aged 40–65 years and to secondarily investigate a possible association with coronary artery calcium score (CACS). **Methods**: This cross-sectional study included 67 men aged 40–65 years referred for coronary computed tomography angiography, sampled in a balanced manner across coronary artery calcium score (CACS) categories. Serum inhibin B levels were measured by a commercial sandwich ELISA; AIP, TyG index, HOMA-IR, and METS-IR were calculated from fasting biochemical measurements. Associations between inhibin B and cardiometabolic parameters were assessed using Spearman correlation analyses and age-adjusted partial correlations. Significant associations were further examined using multivariable linear regression. **Results**: The mean serum inhibin B level was 224 ± 35 pg/mL. The TyG index (ρ = −0.308, *p* = 0.011) and BMI (ρ = −0.348, *p* = 0.004) showed significant negative correlations with inhibin B, and both associations persisted after age adjustment. No significant associations were found with HOMA-IR, METS-IR, AIP, CRP, or CACS. Although BMI and the TyG index were not significantly correlated with each other (ρ = −0.084, *p* = 0.564), both variables remained independently associated with inhibin B in the multivariable model (TyG: β = −0.283, *p* = 0.015; BMI: β = −0.313, *p* = 0.007). The model explained 18.0% of the variance in inhibin B (R^2^ = 0.180). **Conclusions**: In adult men, serum inhibin B levels were independently and inversely associated with BMI and the TyG index, whereas no significant relationship was observed with other metabolic, inflammatory, atherogenic, or coronary calcification markers. These findings suggest that the relationship between inhibin B and cardiometabolic health is not generalizable across all cardiometabolic indices and may be specifically related to the metabolic profile represented by adiposity and the TyG index.

## 1. Introduction

The relationship between male reproductive function and cardiometabolic health has attracted increasing attention in recent years. Testosterone deficiency is known to have a bidirectional relationship with obesity, type 2 diabetes mellitus, and metabolic syndrome; insulin resistance suppresses testosterone synthesis via the hypothalamic–pituitary–gonadal axis, and low testosterone levels in turn reinforce this vicious cycle by promoting dyslipidemia and visceral adiposity [1]. However, while testosterone reflects Leydig cell function, the relationship between Sertoli cells—the testis’s other essential functional component—and cardiometabolic status has been far less investigated.

Inhibin B is a glycoprotein hormone synthesized mainly by Sertoli cells in the testis and exerts negative feedback on pituitary FSH secretion. Because circulating inhibin B reflects germ cell mass and the functional status of spermatogenesis, it serves as an important circulating marker of Sertoli cell function in adult men [2]. Sertoli cells are highly metabolically active cells responsible for maintaining the blood–testis barrier and supplying energy substrate (lactate) to germ cells; it is therefore biologically plausible that systemic metabolic disturbances would affect their function [3].

A limited number of human studies support this biological expectation. Robeva et al. reported that both anti-Müllerian hormone (AMH) and inhibin B levels were significantly lower in men with metabolic syndrome compared with healthy controls, suggesting primary Sertoli cell dysfunction [4]. In a more recent, large-scale NHANES analysis, Zhou and Zhou showed that body mass index was independently and negatively associated with both AMH and inhibin B in adult men, with this relationship being nonlinear and becoming markedly steeper above a BMI threshold of approximately 34 kg/m^2^ [5]. On the coronary artery disease (CAD) front, Kocoglu et al. reported that serum inhibin B levels were significantly lower in older men with angiographically confirmed CAD compared with healthy controls, although no significant correlation was found between inhibin B and Gensini score within the CAD group [6].

A recent animal study showed that insulin resistance suppressed glycolytic enzyme expression (HK2, PKM2, LDHA) in Sertoli cells and increased germ cell apoptosis in diet-induced obese mice; treatment with an insulin-sensitizing compound significantly increased serum inhibin B levels, along with testosterone and FSH. This finding provides experimental mechanistic support for the notion that insulin resistance leads to Sertoli cell dysfunction and that this dysfunction is reversibly reflected in inhibin B levels [3].

Large-scale studies along the testosterone axis have reported that the atherogenic index of plasma (AIP), the metabolic score for insulin resistance (METS-IR), and triglyceride–glucose-based metabolic indices are associated with testosterone levels or testosterone deficiency [7,8,9]. Similarly, the cardiometabolic index (CMI) has been shown to be independently and inversely associated with testosterone levels in adult men [10]. Studies on anti-Müllerian hormone (AMH), another Sertoli cell-derived marker, also suggest that Sertoli cell-derived markers may be related to inflammation and cardiometabolic status [11,12]. Together, these findings point to a shared pathophysiological basis through which cardiometabolic dysregulation affects male gonadal function.

To our knowledge, no human study has jointly evaluated inhibin B against this broad panel of cardiometabolic indices in adult men; the existing evidence is confined to broader measures such as BMI and metabolic syndrome [4,5] or is drawn from the testosterone axis, which reflects Leydig rather than Sertoli cell function [8,9,10]. This gap is not merely descriptive: because AIP, the TyG index, HOMA-IR, METS-IR, and CRP capture partially distinct dimensions of cardiometabolic disturbance—lipid-based atherogenicity, glucose–lipid interaction, insulin sensitivity, and inflammation, respectively—it remains unknown whether Sertoli cell function, as reflected by inhibin B, responds uniformly to cardiometabolic dysregulation or is selectively associated with specific metabolic pathways. We hypothesized that the latter is true: that inhibin B would show a marker-specific rather than a generalized association with cardiometabolic status, in contrast to the broader, more uniform associations reported along the testosterone axis. Testing this hypothesis within a single, well-characterized cohort rather than inferring it indirectly across studies using different markers and populations is the principal contribution of the present study.

The aim of this study was to evaluate the relationships between serum inhibin B levels and body mass index (BMI), atherogenic index of plasma (AIP), triglyceride–glucose (TyG) index, homeostatic model assessment of insulin resistance (HOMA-IR), metabolic score for insulin resistance (METS-IR), and C-reactive protein (CRP) in adult men aged 40–65 years and to secondarily investigate a possible association with coronary artery calcium score (CACS).

## 2. Materials and Methods

### 2.1. Study Design, Setting, and Ethical Approval

This study is a cross-sectional investigation conducted within the Department of Medical Biochemistry, Aydın Adnan Menderes University Faculty of Medicine Hospital. The study protocol was approved by the Aydın Adnan Menderes University Faculty of Medicine Clinical Research Ethics Committee (Approval No.: 2026/278, Date: 9 July 2026) and was conducted in accordance with the principles of the Declaration of Helsinki. Written informed consent was obtained from all participants included in the study, including those whose serum had been collected as part of a related study on alpha-Klotho and cardiometabolic parameters (Approval No.: 2026/93, Date: 2 March 2026), for which the corresponding ethics approval explicitly permitted the use of banked serum in future related studies.

### 2.2. Study Population and Sample

During the study period, adult men referred for clinically indicated coronary computed tomography angiography (CCTA) were evaluated. Based on the coronary artery calcium score (CACS) obtained during the same imaging session, participants were divided into three categories (CACS = 0; CACS 1–299; CACS ≥ 300), and a total of 90 patients were enrolled using balanced sampling with 30 patients per category. The age range was limited to 40–65 years; this upper limit was set to prevent the physiological decline in inhibin B observed with advancing age from confounding the study results.

Participants were drawn from two sources. A subset of 32 participants had blood samples collected between 1 April 2026 and 30 June 2026 as part of a related study on serum alpha-Klotho and cardiometabolic parameters, conducted under a separate ethics approval permitting the use of banked serum in future related studies. The remaining participants were prospectively and consecutively enrolled between 15 July 2026 and 1 August 2026. For all participants, blood samples for biochemical and inhibin B measurements were obtained on the same day as the CCTA examination, following written informed consent, ensuring temporal concordance between the coronary imaging assessment and the biomarker measurements. All biochemical and inhibin B measurements, for both previously collected and newly obtained samples, were performed together on 1 August 2026.

Inclusion criteria:

- Adult men aged 40–65 years, consecutively enrolled among patients referred for clinically indicated coronary computed tomography angiography (CCTA), irrespective of the specific clinical indication for referral (e.g., suspected or known coronary artery disease, chest pain, cardiovascular risk factor screening).
- Provision of written informed consent.
- Sufficient serum volume available for the required biochemical measurements.

Exclusion criteria:

- Known active malignancy.
- Chronic kidney disease (estimated glomerular filtration rate < 30 mL/min/1.73 m^2^).
- Acute infection or active inflammatory disease (given its potential to affect CRP measurement).
- Primary hypogonadism or a history of testicular disease or orchiectomy.
- Chronic liver disease.
- Current statin use.
- Current antidiabetic medication use.
- Cognitive impairment precluding informed consent.

**Figure 1:**
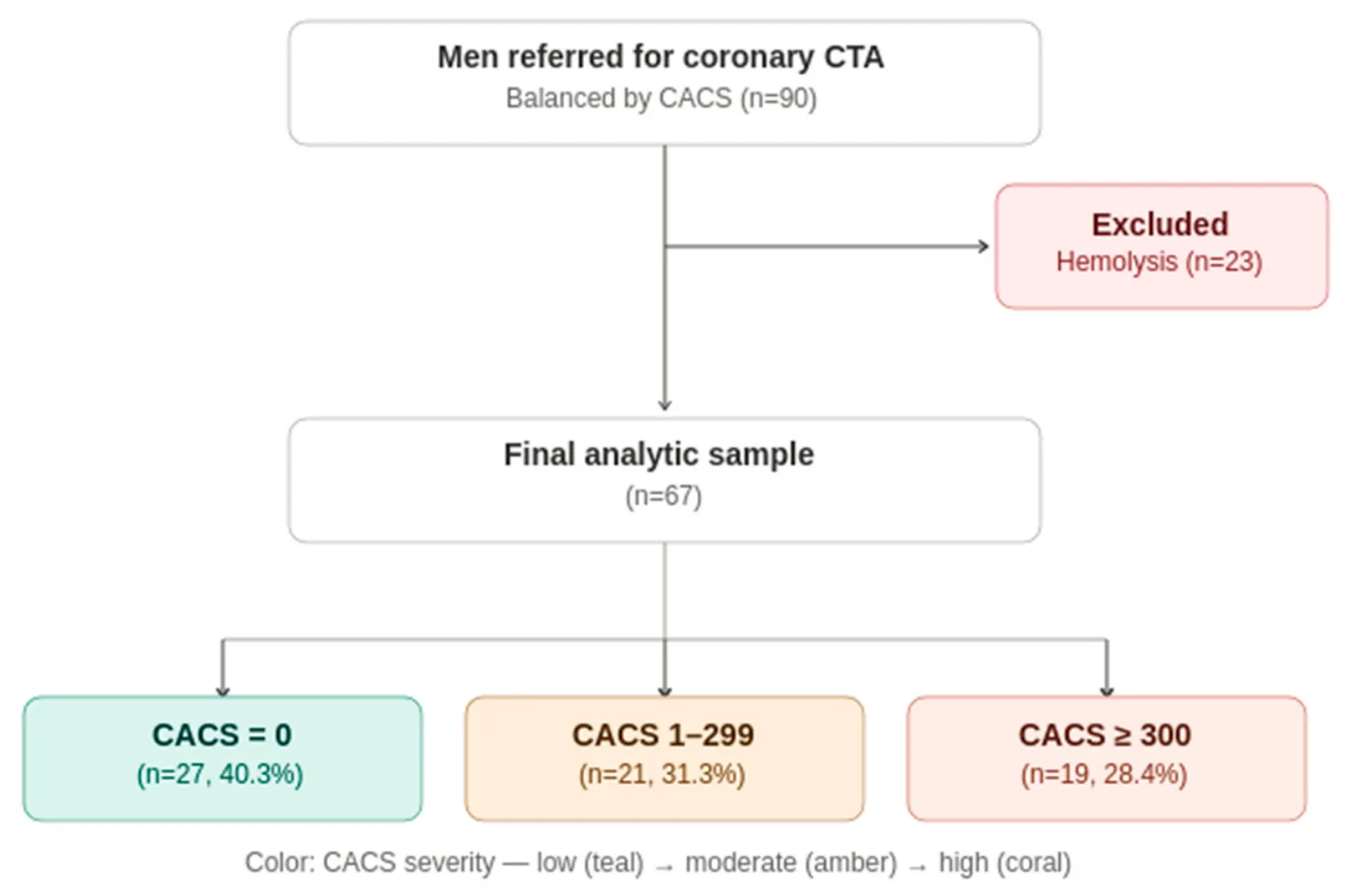
Study Participant Flow Diagram. CCTA: coronary computed tomography angiography; CACS: coronary artery calcium score.

Because some of the collected blood samples could not be reliably analyzed due to hemolysis, the affected patients were excluded from the study, resulting in a final analytic sample of *n* = 67. The loss due to hemolysis was not evenly distributed across CACS categories, and this is addressed separately in the limitations section. Because of missing values for some biochemical parameters (CRP, fasting insulin, etc.), the number of participants for whom the corresponding derived indices (HOMA-IR, AIP, METS-IR) could be calculated varied; the *n* used for each analysis is indicated separately in the relevant table. The participant selection process and the distribution by coronary artery calcium score category are summarized in Figure 1.

### 2.3. Blood Sample Collection and Biochemical Measurements

Peripheral venous blood samples were obtained from participants after an 8–12 h fast. Serum samples were separated by centrifugation and stored at −80 °C until inhibin B measurement.

Fasting glucose, triglyceride, and HDL-cholesterol levels were measured by enzymatic colorimetric methods, and serum CRP level was measured by the immunoturbidimetric method. Fasting insulin was determined by chemiluminescent immunoassay (CLIA). All biochemical parameters were analyzed on the Abbott Alinity autoanalyzer (Abbott Laboratories, Abbott Park, IL, USA).

Serum inhibin B level was measured using a commercial sandwich ELISA kit (Human INHB ELISA Kit, Cat. No. E-EL-H0313, Elabscience, Houston, TX, USA) according to the manufacturer’s instructions. The kit’s analytical sensitivity was 4.69 pg/mL, with a detection range of 7.81–500 pg/mL, and intra-assay and inter-assay coefficients of variation were both reported to be below 10%.

### 2.4. Derived Cardiometabolic Indices

Derived cardiometabolic indices were calculated using the following formulas:

- Atherogenic plasma index (AIP) = log_10_ [Triglyceride (mmol/L)/HDL-cholesterol (mmol/L)] [13].
- Triglyceride–glucose (TyG) index = ln [Triglyceride (mg/dL) × Fasting glucose (mg/dL)/2] [14].
- HOMA-IR = [Fasting glucose (mg/dL) × Fasting insulin (µIU/mL)]/405 [15].
- METS-IR = ln [(2 × Fasting glucose (mg/dL)) + Triglyceride (mg/dL)] × BMI/ln [HDL-cholesterol (mg/dL)] [16].

Because waist circumference data were not available, the visceral adiposity index (VAI) and lipid accumulation product (LAP) could not be calculated and were excluded from analysis. Body mass index (BMI) was included for those participants for whom it was available. BMI was selected as the primary adiposity measure in this study because it is routinely available in clinical practice, standardized, and directly comparable with prior studies examining the relationship between adiposity and Sertoli cell-derived markers, including AMH and inhibin B [4,5].

### 2.5. Coronary CT Angiography Protocol

Prior to contrast-enhanced CCTA, a non-contrast, ECG-gated coronary artery calcium scan was acquired in the same imaging session using prospective (sequential) ECG triggering. Calcium scoring acquisition parameters were as follows: section thickness, 3 mm; tube voltage, 120 kVp; tube current, 250 mA. Coronary artery calcium burden was quantified from these non-contrast images using the standard Agatston method, applying a fixed attenuation threshold of 130 Hounsfield units (HU). All coronary CT angiography examinations were performed using a 160-slice, 128-detector multidetector computed tomography (MDCT) scanner (Aquilion Prime, Toshiba Medical Systems, Otawara, Japan). Iodinated contrast material (iohexol, 350 mg/mL; Omnipaque, GE HealthCare Ireland Limited, Cork, Ireland) was administered via the left antecubital vein at a rate of 4 mL/s (70–100 mL total volume) using an automated injection device. Following the onset of contrast infusion, scanning was performed from the cardiac apex to the base using the bolus tracking technique. All examinations were performed in the supine position within a single breath-hold. Scans were acquired with electrocardiography (ECG)-gated prospective or retrospective modulation; in retrospective modulation, all phases across 0–90% of the R-R interval were reconstructed at 10% intervals.

Imaging parameters were as follows: section thickness, 0.5 mm; rotation time, 400 ms; 300–400 mAs; 100 kVp; section spacing, 0.25 mm. Axial MDCT sections were transferred to a workstation and evaluated using three-dimensional volume rendering, maximum-intensity projection (MIP), and two-dimensional multiplanar reconstructions (MPRs). Vessel segmentation was performed using a dedicated cardiac analysis software (Terarecon-Aquarius Workstation Intuition Edition v.4.4.7.1021.7056); TeraRecon, Inc., Durham, NC, USA).

### 2.6. Statistical Analysis

Statistical analyses were performed using SPSS Statistics 26 (IBM Corp., Armonk, NY, USA), and results were independently verified using Python 3.13.5 (pandas 2.2.3, SciPy 1.17.0, statsmodels 0.14.6). The normality of distribution of continuous variables was assessed by visual inspection and the Shapiro–Wilk test. As serum inhibin B, the common variable across all correlation analyses, did not follow a normal distribution (Shapiro–Wilk *p* = 0.031), Spearman correlation coefficients were used for all correlation analyses. To control for the potential confounding effect of age on the relationship between inhibin B and cardiometabolic parameters, age-adjusted Spearman partial correlations were calculated.

Variables significantly correlated with inhibin B were jointly entered into a multivariable linear regression model; multicollinearity among variables was assessed using the variance inflation factor (VIF). Differences in inhibin B levels among coronary artery calcium score categories were compared using the Kruskal–Wallis test, owing to the right-skewed, zero-inflated distribution. Statistical significance was set at *p* < 0.05.

## 3. Results

### 3.1. Participant Characteristics

A total of 67 male patients meeting the inclusion criteria (40–65 years) were included in the study (the targeted *n* = 90 could not be reached due to samples rendered unusable by hemolysis). The demographic and clinical characteristics of participants are shown in Table 1. Mean age was 56.8 ± 7.7 years (range: 40–65). Mean body mass index (BMI) was 29.0 ± 4.2 kg/m^2^ (range: 22.8–52.1). Serum inhibin B level was 224 ± 35 pg/mL (range: 161–308). Based on coronary artery calcium score (CACS), patients were divided into three categories: CACS = 0 (*n* = 27), CACS 1–299 (*n* = 21, mean 159.9), and CACS ≥ 300 (*n* = 19, mean 836.1).

### 3.2. Relationship Between Inhibin B and Cardiometabolic Parameters

The TyG index and BMI were identified as the two parameters showing significant negative correlations with inhibin B (ρ = −0.308, *p* = 0.011 and ρ = −0.348, *p* = 0.004, respectively). Age-adjusted Spearman partial correlations yielded similar results (ρ = −0.314, *p* = 0.010 and ρ = −0.360, *p* = 0.003, respectively). While no significant correlation was found between the TyG index and BMI, both variables were independently associated with inhibin B in the multivariable model. All correlation results are presented in Table 2.

When these two variables were entered together into a multivariable linear regression model, the model was statistically significant (F(2,64) = 7.017; *p* = 0.002; R^2^ = 0.180; adjusted R^2^ = 0.154), and both variables retained significance as independent predictors in the model. Detailed regression results are shown in Table 3.

### 3.3. Parameters Without Significant Associations

Because no significant relationship was found between inhibin B and age, CRP, HOMA-IR, AIP, or METS-IR, these parameters were not included in the regression model (Table 2). No significant difference in inhibin B level was found among CACS categories either (Kruskal–Wallis H = 1.756, *p* = 0.416) (Table 4).

## 4. Discussion

In this cross-sectional study, serum inhibin B levels were shown to be significantly and negatively associated with the TyG index and body mass index (BMI) in adult men aged 40–65 years. Notably, BMI and the TyG index remained independently associated with inhibin B despite being statistically unrelated to one another (ρ = −0.084, *p* = 0.564), suggesting that adiposity and glucose–lipid dysregulation act on Sertoli cell function through at least partially distinct pathways—a dissociation that, to our knowledge, has not previously been demonstrated for any Sertoli cell-derived marker. These associations persisted after adjustment for age, and both variables retained their independent association with inhibin B when jointly entered into the multivariable model. In contrast, no significant relationship was found between inhibin B and HOMA-IR, METS-IR, the atherogenic index of plasma (AIP), C-reactive protein (CRP), or coronary artery calcium score (CACS). Our findings suggest that the relationship between inhibin B and cardiometabolic health is not generalizable across all cardiometabolic indices and may instead differ according to markers reflecting adiposity and glucose–lipid metabolic burden. To our knowledge, this is among the first studies to jointly evaluate inhibin B in adult men alongside this panel of markers representing different cardiometabolic dimensions.

The negative association we found between BMI and serum inhibin B levels is consistent with existing human data showing a relationship between adiposity and Sertoli cell function. Moreover, when BMI was included in the multivariable model together with the TyG index, it retained its independent negative association with inhibin B. In Zhou and Zhou’s NHANES analysis of 728 adult men, BMI was similarly shown to be independently and negatively associated with inhibin B; this relationship was reported to be nonlinear, with the negative slope becoming more pronounced above a BMI threshold of approximately 33.86 kg/m^2^ [5]. In the present study, the large majority of participants (89.6%; *n* = 60/67) had BMI values below this threshold (mean 29.0 ± 4.2 kg/m^2^), suggesting that the negative association between BMI and inhibin B may exist not only at advanced levels of obesity but also across a more moderate range of adiposity. Similarly, Robeva et al. reported that inhibin B and AMH levels were lower in men with metabolic syndrome compared with healthy controls and that inhibin B was inversely related to BMI [4].

This relationship is further supported by broader evidence. In a meta-analysis evaluating the effects of metabolic syndrome on male reproductive hormones and reproductive function, a pooled analysis of four studies examining inhibin B compared 245 men with metabolic syndrome with 1498 controls and found significantly lower inhibin B levels in the metabolic syndrome group (SMD = −2.95; 95% CI: −5.19 to −0.71). However, the considerably high heterogeneity across studies in this analysis (I^2^ = 99%) indicates that the magnitude of this relationship may vary across different study populations and metabolic phenotypes and that the finding should be interpreted with caution [17]. The BMI–inhibin B relationship observed in the present study supports the notion that increasing adiposity may be associated with inhibin B as a continuous variable, beyond a categorical definition such as metabolic syndrome.

Several biological mechanisms have been proposed for the relationship between obesity and lower inhibin B levels. Insulin resistance accompanying adiposity, chronic low-grade inflammation, hormonal changes, and disturbances in the testicular microenvironment may all affect Sertoli cell function. Experimentally, in diet-induced obese mice, insulin resistance has been shown to suppress the expression of glycolytic enzymes such as HK2, PKM2, and LDHA in Sertoli cells and to increase germ cell apoptosis; improvement in insulin sensitivity was accompanied by an increase in serum inhibin B levels [3]. These findings provide a biologically plausible basis for the observed relationship between adiposity and inhibin B; however, given the cross-sectional design of the present study, no inference can be made regarding the direction or causality of this relationship.

One of the notable findings of our study is the negative relationship observed between the TyG index and serum inhibin B levels. This relationship persisted after adjustment for age, and the TyG index retained its independent association with inhibin B when entered into the multivariable model together with BMI. The TyG index, which combines triglyceride and fasting glucose into a single measure, is widely used as a practical surrogate marker of insulin resistance. Although direct human data evaluating the relationship between the TyG index and inhibin B are limited, there is evidence supporting a relationship between the TyG index and male reproductive function. Belladelli et al. showed that a higher TyG index (≥8.1) was associated with lower total testosterone and sperm concentration, higher sperm DNA fragmentation, and a higher frequency of idiopathic non-obstructive azoospermia in 726 men with primary infertility. The TyG index also correlated significantly with HOMA-IR and retained its independent association with several adverse semen outcomes in multivariable analyses [18]. Although this study did not evaluate inhibin B, it provides indirect clinical support for our findings regarding the relationship between the TyG index and male gonadal and spermatogenic function.

A biologically plausible explanation for the TyG–inhibin B relationship may relate to the distinct energy metabolism of Sertoli cells. Sertoli cells are metabolically active cells that convert glucose largely into lactate to meet the energy requirements of developing germ cells. In the same experimental model referenced above in the context of the BMI–inhibin B relationship, Fu et al. demonstrated in a diet-induced obese mouse model and in TM4 Sertoli cells that insulin resistance was accompanied by decreased expression of glycolytic enzymes such as HK2, PKM2, and LDHA, impaired glycolytic activity, and increased germ cell apoptosis; an improvement in the metabolic disturbance was accompanied by an increase in serum inhibin B levels [3]. These findings point to a biologically plausible link between impaired glucose–lipid metabolism and Sertoli cell function. However, it should be noted that these experimental findings cannot be directly generalized to humans, and the cross-sectional design of the present study does not permit inference regarding the direction or causality of the relationship between the TyG index and inhibin B.

In our study, a significant and independent negative relationship was found between the TyG index and inhibin B, whereas no significant relationship was demonstrated with HOMA-IR or METS-IR. This divergence should be interpreted with the understanding that different insulin resistance markers are not entirely equivalent to one another. HOMA-IR is based on fasting glucose and insulin concentrations, whereas the TyG index is an insulin-independent surrogate marker combining fasting glucose and triglyceride; METS-IR, in turn, is a more composite metabolic index incorporating glucose, triglyceride, HDL-cholesterol, and BMI components. These indices may therefore reflect partially distinct dimensions of cardiometabolic disturbance.

Studies on the testosterone axis in men have also shown that these indices can perform differently. Li and Xu reported that METS-IR was independently and negatively associated with serum testosterone levels and showed higher discriminatory performance than several metabolic markers, including HOMA-IR and TyG, in predicting testosterone deficiency [10]. However, these findings pertain to testosterone, which reflects Leydig cell function, and cannot be directly generalized to inhibin B, a marker of Sertoli cell function. The finding that only the TyG index was associated with inhibin B in the present study suggests that different metabolic indices may not carry the same biological sensitivity with respect to Sertoli cell function. Furthermore, although no significant correlation was found between BMI and the TyG index, both variables retained their independent association with inhibin B in the multivariable model, suggesting that the metabolic profiles represented by adiposity and the TyG index may carry partially distinct information in relation to inhibin B. However, this finding does not prove that these relationships operate through distinct pathophysiological mechanisms.

On the other hand, the absence of a significant relationship for HOMA-IR and METS-IR should not be interpreted as indicating that insulin resistance is unrelated to inhibin B. In particular, the smaller number of participants in the METS-IR analysis (*n* = 51) may have reduced the likelihood of detecting weaker associations. Furthermore, given the cross-sectional nature of the study and the fact that these indices rely on distinct biochemical components, the specific mechanism underlying the TyG–inhibin B relationship cannot be determined from the present data. Our findings therefore indicate that the metabolic profile represented by the TyG index rather than a general “insulin resistance–inhibin B” relationship is associated with lower inhibin B levels.

No significant relationship was found between AIP and serum inhibin B levels in our study. This finding differs in part from the predominantly testosterone-based literature examining the relationship between male gonadal function and cardiometabolic markers. Tai et al., in their NHANES-based study, reported that elevated AIP was independently and positively associated with testosterone deficiency [9]. The broader cardiometabolic literature has also shown that AIP is consistently associated with triglyceride, HDL-cholesterol, and insulin resistance but that its relationship with certain cardiovascular parameters such as blood pressure remains inconsistent, indicating that AIP may not be an equally strong predictor across every physiological axis (Lioy et al.) [19]. However, testosterone reflects Leydig cell function, whereas inhibin B predominantly reflects Sertoli cell function and spermatogenic activity—distinct biological markers. The AIP–testosterone relationship therefore need not necessarily hold true for inhibin B as well. The absence of a relationship between AIP and inhibin B in the present study does not prove that the atherogenic lipid profile has no direct, measurable relationship with Sertoli cell function, but it does suggest that cardiometabolic relationships demonstrated along the testosterone axis cannot be directly generalized to inhibin B.

Similarly, no significant relationship was found between serum CRP levels and inhibin B. In contrast, Kadariya et al. reported an independent, inverse relationship between AMH—another Sertoli cell-derived hormone—and CRP in adult men [12]. However, a comprehensive systematic review evaluating the relationship between AMH and cardiometabolic status found this relationship to be inconsistent across studies, with no significant association demonstrated for several parameters [20]. Because AMH and inhibin B, although secreted from the same cellular source, are subject to different physiological regulators and exhibit different secretion patterns across the lifespan, inflammation-related findings derived from AMH should not be directly extrapolated to inhibin B. Among the limited direct human data on the inflammation–inhibin B relationship, Al-Bashiti et al. reported that serum inhibin B levels showed a significant negative correlation with TNF-α in adult men with COVID-19 (r = −0.326, *p* = 0.003) [21]. However, CRP was not assessed in that study, and given the distinct inflammatory milieu specific to COVID-19, these findings cannot be directly generalized to the present study population. Taken together, these findings suggest that the relationship between systemic inflammation and inhibin B remains unclear in adult men and that larger studies evaluating CRP alongside other inflammatory markers are warranted.

No significant relationship was found between coronary artery calcium score and serum inhibin B levels in our study, either in the continuous analysis or across CACS categories. This finding is partly consistent with results reported by Kocoglu et al. in older men with coronary artery disease. In that study, serum inhibin B levels were significantly lower in men with angiographically confirmed coronary artery disease compared with healthy controls; however, no significant correlation was found between inhibin B and Gensini score within the coronary artery disease group [6]. This suggests that a possible relationship between the presence of coronary artery disease and inhibin B may not necessarily translate into a linear relationship with the degree of atherosclerotic burden.

It should nonetheless be considered that the Gensini score and coronary artery calcium score assess different features of atherosclerosis. The Gensini score evaluates the angiographic severity of coronary luminal narrowing, whereas CACS reflects calcified coronary plaque burden. The results of the two studies are therefore not directly comparable. The absence of a relationship between inhibin B and CACS in the present study suggests that circulating inhibin B levels may not be a marker reflecting the degree of subclinical coronary calcification burden; however, this result should be interpreted cautiously given the sample size and cross-sectional design. Because participants were initially sampled in a balanced manner across CACS categories (30 patients per category), the CACS groups were relatively evenly represented in the comparison, which increases the likelihood that the observed lack of association reflects a true absence of relationship rather than insufficient statistical power.

The main strength of this study is its joint evaluation of serum inhibin B levels in adult men alongside AIP, the TyG index, HOMA-IR, METS-IR, CRP, and coronary artery calcium score markers representing different dimensions of cardiometabolic health. In addition, participants were sampled in a balanced manner across coronary artery calcium score categories, which reduced the risk of an uneven distribution of atherosclerotic burden across the sample and allowed for a more representative comparison of inhibin B across the full spectrum of subclinical coronary calcification. Given that the existing literature has largely focused on the testosterone axis or on broader measures such as BMI and metabolic syndrome, our study offers a complementary perspective through inhibin B as a marker of Sertoli cell function. Furthermore, the persistence of the associations between the TyG index and BMI with inhibin B after age adjustment and the fact that both variables retained their independent association with inhibin B in the multivariable model support the consistency of our findings.

Nonetheless, this study has several limitations. First, the cross-sectional design does not allow inference regarding the direction or causality of the observed associations. Due to hemolysis, the targeted 90 participants could not be reached, and the final sample was limited to 67 participants; furthermore, the sample size analyzed varied due to missing data for some parameters, with the METS-IR analysis in particular being conducted with only 51 participants. Although participants were initially sampled in a balanced manner across CACS categories (30 patients per category), hemolysis-related sample loss was not evenly distributed among categories (CACS = 0: 30→27; CACS 1–299: 30→21; CACS ≥ 300: 30→19), somewhat reducing representativeness particularly in the high-CACS group. Participants with current statin or antidiabetic medication use were excluded, which reduces the risk of pharmacological confounding of the lipid- and glucose-based indices (AIP, TyG index, HOMA-IR, METS-IR) used in this study; nonetheless, information on other medications with potential metabolic effects was not systematically recorded. Similarly, other common comorbidities, such as hypertension, were not captured in this dataset and could therefore not be evaluated as potential confounders. The single-center design may limit the generalizability of the results. The lack of assessment of complementary gonadal hormones such as FSH, testosterone, and AMH, as well as sperm parameters and testicular volume, precludes interpretation of the inhibin B findings alongside other components of gonadal function. Because waist circumference data were unavailable, additional adiposity indices such as the visceral adiposity index and lipid accumulation product could not be evaluated. Furthermore, BMI is an indirect measure of adiposity and does not distinguish fat mass from lean or muscle mass; a high BMI may therefore reflect increased muscle mass rather than excess adiposity in some individuals, which should be considered when interpreting the BMI–inhibin B association reported here.

In light of these limitations, confirmation of these findings in larger, independent populations is warranted, along with prospective studies evaluating inhibin B alongside FSH, testosterone, AMH, semen parameters, and direct adiposity measurements. Longitudinal designs would allow determination of whether changes in cardiometabolic status parallel changes in inhibin B levels over time.

In conclusion, in this study, serum inhibin B levels in adult men were independently and negatively associated with BMI and the TyG index, whereas no significant relationship was found with HOMA-IR, METS-IR, AIP, CRP, or coronary artery calcium score. These findings suggest that the relationship between inhibin B and cardiometabolic health is not generalizable across all metabolic and vascular markers and that this relationship may be more pronounced specifically in relation to the glucose–lipid metabolic profile represented by adiposity and the TyG index. Our findings contribute a Sertoli cell-function perspective to the relationship between cardiometabolic health and gonadal function in men.

## 5. Conclusions

In this cross-sectional study of adult men aged 40–65 years, serum inhibin B levels were independently and negatively associated with the TyG index and BMI, whereas no significant relationship was observed with HOMA-IR, METS-IR, AIP, CRP, or coronary artery calcium score. These findings indicate that the relationship between inhibin B and cardiometabolic health is not uniform across all metabolic and vascular indices but appears to be more closely linked to adiposity and the glucose–lipid metabolic burden captured by the TyG index. To our knowledge, this is among the first studies to evaluate serum inhibin B in relation to this panel of cardiometabolic indices in adult men, offering a Sertoli cell-function perspective that complements the predominantly testosterone-centered literature. These findings should be confirmed in larger, prospective studies incorporating complementary gonadal hormones and direct adiposity measures.

## Figures and Tables

**Table 1:** Participant Characteristics (*n* = 67).

Variable	*n*	Value
Age (years), mean ± SD	67	56.8 ± 7.7
Age range, years	67	40–65
BMI (kg/m^2^), mean ± SD	67	29.0 ± 4.2
BMI range, kg/m^2^	67	22.8–52.1
Serum inhibin B (pg/mL), mean ± SD	67	224 ± 35
Inhibin B range, pg/mL	67	161–308
TyG index, mean ± SD	67	8.89 ± 0.53
CRP (mg/L), mean ± SD	61	0.36 ± 0.73
CACS = 0, *n* (%)	27	40.3%
CACS 1–299, *n* (%)—mean CACS	21	31.3%–159.9
CACS ≥ 300, *n* (%)—mean CACS	19	28.4%–836.1

BMI: body mass index; TyG: triglyceride–glucose index; CRP: C-reactive protein; CACS: coronary artery calcium score. Values are presented as mean ± standard deviation or *n* (%).

**Table 2:** Correlations Between Inhibin B and Cardiometabolic Parameters.

Variable	*n*	Spearman ρ (*p*)	Age-Adjusted ρ (*p*)
TyG index	67	−0.308 (0.011) *	−0.314 (0.010) *
BMI	67	−0.348 (0.004) *	−0.360 (0.003) *
Age	67	0.051 (0.680)	—
CRP	61	−0.083 (0.527)	—
HOMA-IR	65	0.155 (0.216)	—
AIP	61	0.157 (0.227)	—
METS-IR	51	−0.151 (0.291)	—
CACS (raw score)	67	0.103 (0.406)	—

* *p* < 0.05. Correlations were assessed using Spearman correlation coefficients, as serum inhibin B did not follow a normal distribution (Shapiro–Wilk *p* = 0.031). Age-adjusted values represent Spearman partial correlations controlling for age. AIP: atherogenic index of plasma; TyG: triglyceride–glucose index; HOMA-IR: homeostatic model assessment of insulin resistance; METS-IR: metabolic score for insulin resistance; CRP: C-reactive protein; CACS: coronary artery calcium score.

**Table 3:** Multiple Linear Regression Model Predicting Inhibin B.

Variable	B	SE	β	t	*p*	95% CI
Constant	463.08	70.95	—	6.527	<0.001	321.4–604.8
TyG index	−18.47	7.40	−0.283	−2.497	0.015	−33.3 to −3.7
BMI	−2.58	0.93	−0.313	−2.766	0.007	−4.5 to −0.7

Dependent variable: serum inhibin B (pg/mL). Model: F(2,64) = 7.017; *p* = 0.002; R^2^ = 0.180; adjusted R^2^ = 0.154. VIF (TyG index, BMI) ≈ 1.00—no multicollinearity issue. SE: standard error; β: standardized coefficient; CI: confidence interval.

**Table 4:** Inhibin B Levels by Coronary Artery Calcium Score Category.

CACS Category	*n*	Inhibin B (pg/mL), Mean ± SD	Median
CACS = 0	27	219 ± 41	205
CACS 1–299	21	227 ± 29	223
CACS ≥ 300	19	228 ± 32	221

Kruskal–Wallis H = 1.756; *p* = 0.416 (no significant difference between groups).

## Data Availability

The data that support the findings of this study are available from the corresponding author upon reasonable request.

## Abbreviations

The following abbreviations are used in this manuscript:

**AIP**	Atherogenic index of plasma
**AMH**	Anti-Müllerian hormone
**BMI**	Body mass index
**CACS**	Coronary artery calcium score
**CAD**	Coronary artery disease
**CCTA**	Coronary computed tomography angiography
**CI**	Confidence interval
**CLIA**	Chemiluminescent immunoassay
**CMI**	Cardiometabolic index
**CRP**	C-reactive protein
**ELISA**	Enzyme-linked immunosorbent assay
**FSH**	Follicle-stimulating hormone
**HDL**	High-density lipoprotein
**HOMA-IR**	Homeostatic model assessment of insulin resistance
**HU**	Hounsfield unit
**MDCT**	Multidetector computed tomography
**METS-IR**	Metabolic score for insulin resistance
**MIP**	Maximum-intensity projection
**MPR**	Multiplanar reconstruction
**NHANES**	National Health and Nutrition Examination Survey
**SD**	Standard deviation
**SE**	Standard error
**SMD**	Standardized mean difference
**TG**	Triglyceride
**TNF-α**	Tumor necrosis factor-alpha
**TyG**	Triglyceride–glucose (index)
**VIF**	Variance inflation factor
